# Interrupting the Psychedelic Experience Through Contextual Manipulation to Study Experience Efficacy

**DOI:** 10.1001/jamanetworkopen.2024.22181

**Published:** 2024-07-15

**Authors:** Leor Roseman, David Erritzoe, David Nutt, Robin Carhart-Harris, Christopher Timmermann

**Affiliations:** 1 Department of Psychology, University of Exeter, United Kingdom; 2 Centre for Psychedelic Research, Imperial College London, United Kingdom; 3 School of Medicine, University of California San Francisco

## Abstract

This quality improvement study investigates the association of interruptions during psychedelic therapy with ratings of intensity of experience.

## Introduction

Under the psychedelic therapy paradigm,^1^ instead of looking at drug efficacy, researchers look at *experience efficacy*, defined as how certain experiences can be therapeutic. To test experience efficacy, researchers need to develop new research tools to manipulate the experience without changing the pharmacology.

We suggest that intentional cognitive interruptions can help inquire into experience efficacy by experimentally interfering with the experience. To strengthen this suggestion, we present a secondary analysis from our 2023 N,N-dimethyltryptamine (DMT) study,^2^ investigating whether it is possible to interrupt the psychedelic experience by increasing cognitive load and whether an interrupted experience is associated with reduced long-term mental health changes. We hypothesized that subjective ratings of the psychedelic experience would be lower when task demands were higher and reductions in long-term depressive symptoms would be larger with fewer task demands during the experience.

## Methods

This quality improvement study was approved by the National Research Ethics Committee London–Brent and Health Research Authority and is reported following the SQUIRE reporting guideline. Participants provided informed consent (eMethods in Supplement 1). Healthy volunteers participated in 2 testing days separated by 2 weeks. On each testing day, participants underwent 2 separate intravenous DMT or placebo administrations while being scanned with combined functional magnetic resonance imaging and electroencephalography in a randomized fashion.^2^ In the rating scan, participants were asked to verbally rate the subjective intensity of drug effects every minute in real time, while the no rating scan had no ratings and participants merely rested. Therefore, we were able to assess the association of interruptions from the rating procedure with the DMT experience.

Subjective ratings of the experience were measured retrospectively using the 11 Dimensions Altered States of Consciousness (11D-ASC) questionnaire^3^ after drug effects subsided. The Quick Inventory of Depressive Symptomatology (QIDS) was measured 1 day before and 2 weeks after DMT.

## Results

The analysis included 20 volunteers (mean [SD] age, 33.5 [7.9] years; 7 female [35.0%]) based on previous publications.^2,4^ Using the total ASC score, we confirmed that the total mean (SD) ASC score was higher for the no rating (0.36 [0.08]) than rating (0.29 [0.10]) condition (*P* < .001; within-participant paired *t* test). In further exploratory analysis of 11D-ASC factors, not all factors had equal differences by rating group. Factors previously identified as moderators of clinical efficacy^5,6^ had higher scores in the no rating condition (Figure).

**Figure.**
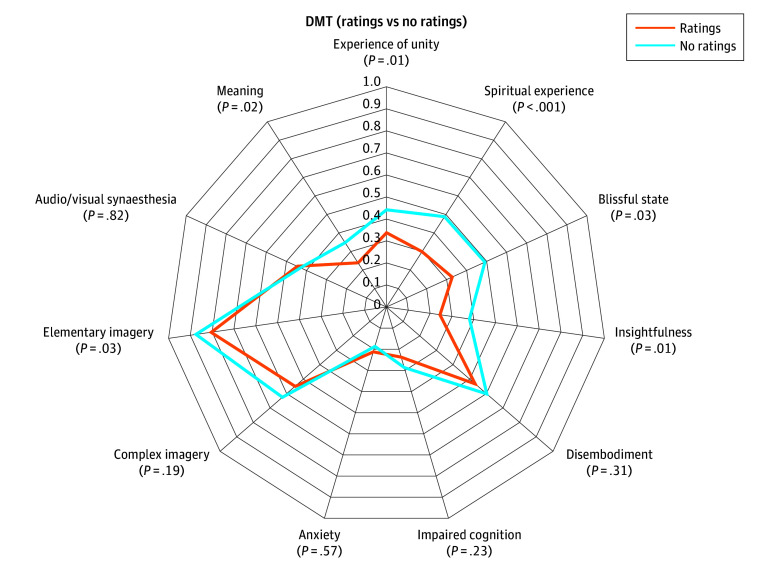
Interruption of Psychedelic Experience and Subjective Intensity Participants underwent 2 scans under the influence of N,N-dimethyltryptamine (DMT) while lying with eyes closed, performing ratings of subjective intensity every 60 seconds in 1 scan. Subjective ratings were measured retrospectively using the 11 Dimensions Altered States of Consciousness questionnaire (eMethods in Supplement 1). With the exception of elementary imagery, significantly diminished dimensions of experience were those previously found to be associated with therapeutic outcomes of psychedelic therapy (experience of unity, spiritual experience, blissful state, insightfulness, and changed meaning of percepts).

We further tested if decreases in depressive symptom ratings after DMT were larger for the no rating condition. To avoid carryover effects, we looked only at changes after the first scan and compared the 12 participants who received DMT first in the rating condition with 8 participants who received DMT first in the no rating condition. Repeated-measure analysis of variance was used to test for an interaction between time (before vs after DMT) and group (rating vs no rating). An interaction was observed for group × time [*F*_1,18_ = 5.9; *P* = .03; ηp^2^ = 0.247], with a greater mean (SD) reduction in QIDS after the no rating (−1.25 [1.04]) than rating (−0.17 [0.94]) scan (*P* = .01). No significant differences were observed for baseline mean (SD) QIDS measures of the no rating (3.12 [1.46]) compared with the rating (1.92 [1.78]) group (*P* = .12; unpaired *t* test).

## Discussion

This quality improvement study’s results suggest that contextual manipulation, such as cognitive interruption (via giving subjective ratings in this study), may be used to study experience efficacy. Conclusions are limited by a small sample size, relatively mild interruption, and status as a secondary analysis of data not intended to test this hypothesis.

For future studies, we suggest that an intentional interruption should be more cognitively demanding than merely asking for ratings. We hope that future psychedelic studies will include contextual manipulation as a procedure to examine safety and efficacy.

## Notes

Supplement 1.
eMethods.

eReferences.

Supplement 2.
Data Sharing Statement
