# Supplementary material for: Interrupting the Psychedelic Experience Through Contextual Manipulation to Study Experience Efficacy
Source: JAMA Netw Open. 2024 Jul 15;7(7):e2422181. doi: 10.1001/jamanetworkopen.2024.22181 (PMC11250380; doi:10.1001/jamanetworkopen.2024.22181)
Supplement: Supplement 1. — eMethods. eReferences. [file jamanetwopen-e2422181-s001.pdf]

Supplemental Online Content

Roseman L, Erritzoe D, Nutt D, Carhart-Harris R, Timmermann C. Interrupting the psychedelic experience through contextual manipulation to study experience efficacy. *JAMA Netw Open*. 2024;7(7):e2422181. doi:10.1001/jamanetworkopen.2024.22181

eMethods.

eReferences.

This supplemental material has been provided by the authors to give readers additional information about their work.

eMethods.

In the secondary analysis presented in this paper, we present the differences in experience and outcome for subjects who received DMT (a 5-HT2A agonist) with or without online ratings during the experience. We were interested in studying whether online verbal ratings (enhanced cognitive demands) will reduce the intensity of the psychedelic experience and impact long-term mental health outcomes.

This study was approved by the National Research Ethics Committee London—Brent and the Health Research Authority and was conducted under the guidelines of the revised Declaration of Helsinki (2000), the International Committee on Harmonization Good Clinical Practices guidelines, and the National Health Service Research Governance Framework. Imperial College London sponsored the research, which was conducted under a Home Office license for research with Schedule 1 drugs.

The original study was a single-blind, placebo-controlled, counter-balanced design[1]. Data was collected from August 2017 to January 2019. An initial screening visit to the Imperial College Research Facility was focused on assessing physical and mental health to ensure suitability. Exclusion criteria included: <18 y old at the moment of participation, MR contraindications, absence of experience with a psychedelic, an adverse reaction to a psychedelic, history of psychiatric or physical illness rendering unsuitable for participation (i.e., diabetes, epilepsy, or heart disease), family history of psychotic disorder, or excessive use of alcohol or drugs of abuse. All participants provided written informed consent for participation in the initial study, which also applies to the current analysis.

Twenty healthy volunteers (mean age = 33.5 y, SD = 7.9, 7 females) participated in two testing days at the Imperial College Clinical Imaging Facility, separated by two weeks. Data was collected originally from twenty-five participants, with five being excluded for various reasons: one participant was excluded due to excessive motion in the scanner; one participant dropped out due to discomfort being inside the scanner for the total duration of the experiment (28 min); two participants failed to show up (adverse reactions were not observed in these cases and unpleasant effects of DMT were not

© 2024 Roseman L et al. *JAMA Network Open.*

mentioned as the cause for dropping out); and one participant only partially completed the study questionnaires.

On each testing day, participants arrived and were tested for drugs of abuse and were involved in two separate scanning sessions. In this initial session (task free, no-ratings), they received intravenous (IV) administration of either placebo (10 mL of sterile saline) or 20 mg DMT (in fumarate form dissolved in 10 mL of sterile saline)—injected over 30 s, and then flushed with 10 mL of saline over 15 s—in a counter-balanced order (half of the participants received placebo and the other half received DMT). The dose was chosen based on our previous dose-finding pharmacokinetic EEG study[2]. This dose is considered a high dose, yielding high subjective intensity ratings.

This first session always consisted of continuous resting-state scans which lasted 28 min with DMT/placebo administered at the end of 8th min and scanning was over 20 min after injection (No-Rating condition). Participants laid in the scanner with their eyes closed (an eye mask was used to prevent eyes opening), while EEG activity was recorded. A second session then followed with the same procedure as the initial session (including scanning conditions), except on this occasion participants were (audio) cued to verbally rate the subjective intensity of drug effects every minute in real-time while in the scanner (Rating condition). By comparing the Rating condition to the No-Rating condition, we were able to assess the impact of the rating procedure on the interruption of the DMT experience.

Following each scanning procedure, participants were interviewed and completed questionnaires designed to assess the subjective effects experienced during the scan [11 Dimensions Altered States of Consciousness Questionnaire—ASC-11D[3]]. The 11D-ASC is a measure to assess altered states of consciousness, which are transient deviations from an individual's normal waking consciousness and has been widely used to measure the acute effects of psychedelic drugs. The total ASC score is calculated as the average of 94 items and presented in percentile from 0 to 1. The scale consists of 11 subscales (i.e., Experience of Unity, Spiritual Experience, Blissful State, Insightfulness, Disembodiment, Impaired Control and Cognition, Anxiety, Complex Imagery, Elementary Imagery, Audio-Visual

© 2024 Roseman L et al. *JAMA Network Open.*

Synaesthesia, and Changed Meaning of Percepts). Our previous work studying psychedelic-assisted therapy for depression showed that dimensions of the ASC, which are similar to peak or mystical-type experiences (unity, spiritual, blissful and insightfulness), are predictors of clinical outcomes[4]. Similar experiences have been shown to be predictors of clinical outcomes in many other psychedelic trials[5].

To give a sense of the different dimensions measured by the ASC, example items for each dimension are presented. Experience of Unity: "I felt one with my surroundings", Spiritual Experience: "I felt full of awe", Blissful State: "I experienced a profound inner peace", Insightfulness: "I had particularly inventive ideas", Disembodiment: "I felt like I was floating", Impaired Control and Cognition: "My thoughts were always interrupted; I could not think anything to its end", Anxiety: "I had the feeling that something terrible was going to happen", Complex Imagery: "I could see images from my memory or imagination with exceeding clarity", Elementary Imagery: "I saw regular patterns in complete darkness or with closed eyes", Audio-Visual Synaesthesia: "Colors seemed to be altered by sounds or noises", and Changed Meaning of Percepts: "Things in my surroundings had a new or alien meaning".

The self-rated 16-item Quick Inventory of Depressive Symptomatology (QIDS) was measured before DMT (1 day prior to the first dosing session) and 2 weeks after the first dosing session (range 12–15 days). QIDS was chosen for this analysis based on previous work from this study[6]. While the population of the study is healthy volunteers, they still showed signs of some depressive symptoms at baseline. To avoid carry-over effects, we looked only at changes after the first scan, and compared the group of subjects who received DMT first in the Rating condition (N=12) against those who received DMT first in the No-Rating condition (N=8). Repeated measure ANOVA was used to test for an interaction between time (before versus after DMT) and group (Rating vs No-Ratings).

This methods section follows the SQUIRE reporting guidelines for Quality improvement (QI) studies[7].

© 2024 Roseman L et al. *JAMA Network Open.*

REFERENCES

1. Timmermann C, Roseman L, Haridas S, et al. Human brain effects of DMT assessed via EEG-fMRI. *Proceedings of the National Academy of Sciences*. 2023;120(13):e2218949120.
2. Eckernäs E, Timmermann C, Carhart-Harris R, Röshammar D, Ashton M. Population pharmacokinetic/pharmacodynamic modeling of the psychedelic experience induced by N, N-dimethyltryptamine–Implications for dose considerations. *Clinical and translational science*. 2022;15(12):2928-2937.
3. Studerus E, Gamma A, Vollenweider FX. Psychometric evaluation of the altered states of consciousness rating scale (OAV). *PLoS One*. 2010;5(8):e12412. doi:10.1371/journal.pone.0012412
4. Roseman L, Nutt DJ, Carhart-Harris RL. Quality of acute psychedelic experience predicts therapeutic efficacy of psilocybin for treatment-resistant depression. *Frontiers in Pharmacology*. 2017;8:974. doi:https://doi.org/10.3389/fphar.2017.00974
5. Kangaslampi S. Association between mystical-type experiences under psychedelics and improvements in well-being or mental health–A comprehensive review of the evidence. *Journal of Psychedelic Studies*. 2023;7(1):18-28.
6. Timmermann C, Zeifman RJ, Erritzoe D, Nutt DJ, Carhart-Harris RL. Effects of DMT on mental health outcomes in healthy volunteers. *Scientific Reports*. 2024;14(1):3097.
7. Ogrinc G, Mooney S, Estrada C, et al. The SQUIRE (Standards for QUality Improvement Reporting Excellence) guidelines for quality improvement reporting: explanation and elaboration. *BMJ Quality & Safety*. 2008;17(Suppl 1):i13-i32.

© 2024 Roseman L et al. *JAMA Network Open.*
