# Supplementary material for: Interrupting the Psychedelic Experience Through Contextual Manipulation to Study Experience Efficacy
Source: JAMA Netw Open. 2024 Jul 15;7(7):e2422181. doi: 10.1001/jamanetworkopen.2024.22181 (PMC11250380; doi:10.1001/jamanetworkopen.2024.22181)
Supplement: Supplement 2. — Data Sharing Statement [file jamanetwopen-e2422181-s002.pdf]

Data Sharing Statement

Roseman. Interrupting the Psychedelic Experience Through Contextual Manipulation to Study Experience Efficacy. *JAMA Netw Open*. Published online July 15, 2024. doi:10.1001/jamanetworkopen.2024.22181

Data
Data available: Yes
Data types: Deidentified participant data **How to access data:** l.roseman@exeter.ac.uk **When available:** With publication

Supporting Documents
Document types: None

Additional Information
Who can access the data: anyone requesting the data
Types of analyses: for any purpose
Mechanisms of data availability: with investigator support
